# A Lecture on Sanguineous Tumours, Delivered at the Hotel Dieu, Paris, May 23d, 1843

**Published:** 1843-09-30

**Authors:** 

**Affiliations:** Paris


THE MEDICAL EXAMINER,

And Retrospect of the Medical Sciences.

Vol. VI.] PHILADELPHIA, SATURDAY, SEPTEMBER 30, 1843. [No. 19.

### A LECTURE ON SANGUINEOUS TUMOURS

*Delivered at the Hotel Dieu, Paris, May 3d.*, 1843.

BY M. ROUX.

Fungous or varicose sanguineous tumours, considered in a general point of view, present some analogy to aneurisms, and it is sometimes difficult to distinguish in this respect, what are the resemblances between these two affections, which are regarded as so essentially different? As you advance in your profession, become familiar with it practically, and your observation of morbid structures increases, you will recognize this principle, viz., that diseases have never naturally the same well-defined characters that you would expect to find in them, after reading their description in the dogmatical treatises. On the contrary, you will constantly find characters which approach them, and even link them together, so that we may say, what I have often remarked to you before, that there exists a great link in the science of human suffering, studied in nature. Thus, for example, in the fungous sanguine tumours you will find certain characters belonging to cancer. Besides the areolar and spongy texture belonging to these tumours, you observe a tissue somewhat melanic, liable to frequent and copious hemorrhages, terminating frequently in death; *vice versa* in tumours essentially cancerous, you often find characters belonging to other diseases, and particularly to sanguineous tumours, such as well marked arterial beating, frequent hemorrhages, a spongy tissue, &c., to such a degree indeed, that the most skilful practitioners are often deceived, and entirely misunderstand the true nature of the disease.

I recollect very well a foreign lady to whom I was called, during one of my voyages, a long time ago. She had upon the foot a tumour, which the surgeons of the country believed to be purely sanguineous, but which I thought was cancerous. I ventured even to propose an operation, which was acceded to and performed by myself. The patient recovered entirely. On examination the tumour presented characters common to the two affections.

The English call a *fungus hematodes*, a tumour which is not cancerous, but which has many characters belonging to those affections, as well as others attaching to tumours of a sanguineous nature. This is a sanguineous tumour entirely peculiar. In France we first accepted this new name, but, on better examining the disease, we found it to be only a sanguineous tumour, where the fungoid element predominated, and that it was useless to introduce into the science a new Greek-Latin name, to designate it. I myself believed, in particular, that no other denomination suited it better than that of fungoid tumour; for by it is better conveyed the idea of a fungous element, which is characteristic of the disease.

To come now to the points of resemblance which exist between sanguineous tumours proper, and aneurisms. Among the most simple tumours you will find many to consist in a considerable developement of the arteries in the neighbourhood, including the vascular capillaries. In aneurisms, on the contrary, there is an abnormal dilatation of one of the large vascular trunks, very often the principal vessel of the region; thus, in the two diseases there is considerable abnormal developement of the vessels. Now for the analogy; in one the dilatation is confined to the capillaries; in the other it is a large vessel which is ordinarily enlarged; behold the difference. Some tumours are formed by the slow extravasation of blood escaping from the vessels, and mingling with the tissues in which it is effused to such a degree, as to form a mass; this is happily rare, for these tumours are of the gravest kind. Sometimes the walls of the vessels become penetrated with little holes, through which the blood oozes slowly, infiltrating the surrounding tissues, which imbibe it, and form fungous areolar tumours of a venous or arterial nature. This variety of tumours was first described by Pott, who, meeting with tumours in the popliteal region, which he at first considered of an aneurismal nature, found, after having dissected them, that he was mistaken, for that they did not positively present the characters of aneurism; and this great surgeon, in the exact description which he gave, distinguished very ably the difference between tumours apparently aneurismal, and ordinary aneurisms. Pelletan, in his Surgical Clinic, cites also some remarkable cases of tumours of this kind. What was said of arteries being sometimes found with holes through which the blood oozed and infiltrated the tissues, can be applied to the veins, whose walls are sometimes subject to the same alterations, and the blood escaping into the tissues, forms venous tumours of the same nature. There is, then, amongst all these affections, certain relations, which in a philosophic point of view, make us consider them as belonging all to the same family. The ordinary sanguine tumour results, as I have said, from the dilatation of a part of the vascular system; that is to say, of the vessels which occupy a particular region. They commence ordinarily by a dilatation of the vascular capillaries of this region; a dilatation which gradually and consecutively extends to the deeper seated vessels; and hence the developement of the tumour.

These tumours have received different names of late years; but I consider them all as more or less inexact, and think that of sanguine tumour is the best. Dupuytren called them erectile tumours, on account of their peculiar elastic nature, which name was adopted by his students and is still employed by a great number of surgeons of the present day. In spite of the great respect in which I hold the memory of this skilful surgeon, I own to you frankly that I consider this name as the worst of all. For, if it suits somewhat tumours of an arterial nature, in which this state of erithism is principally observed, it is entirely inapplicable to the venous variety which does not present this character. Besides, what comparison can be rigidly made between the erithism of these tumours, and the erection of the genital organs, as the penis, clitoris, nipple, &c.? In these latter organs it is a physiological functional condition that we have to do with, whilst in sanguine lumours it is the pathological state of the vessels. Hence this denomination is bad under a philosophical point of view. For me the old name of fungous tumours, although not too precise, and not very scientific, is better, inasmuch as it gives us the prominent and essential features of the disease.

These tumours differ among themselves. 1st. As to their origin; some of them being developed in structures where nothing abnormal existed which could lead to the suspicion of the formation of the disease, and are produced under the influence of causes altogether inappreciable; others are formed under the influence of a pre-existing abnormal state; as, for example, the little spots on the skin constituting a large family of deformities, known as *nævi materni*. There are many kinds of nævi. There are some which necessarily, sooner or later, must give place to fungous tumours. Thus, to give you an example which you have lately had under your eyes, the little child that I recently cut for stone, has, a little above the exterior angle of the left eye a small rose spot, somewhat *piquetée*, which is slightly elevated above the surrounding skin, which certainly sooner or later will give rise to a fungous tumour, if means are not taken to ensure its removal.

The developement of these tumours consecutive to *nævi materni* is sometimes slow, sometimes speedy. Certain periods of life seem to exercise a certain influence over them; thus it is a fact confirmed by observation that the approach of puberty in young girls is a cause which greatly contributes to the developement of these tumours. There is a remarkable case in support of this statement published in the *Clinique Chirurgicale* of Pelletan, and which I was enabled to follow in all its varieties, in the service of that surgeon.

Ordinarily, or very often, these congenital marks are seen on the superior portion of the body, that is upon the face, the neck, arms, &c. They are very often multiple. I have removed four sanguine tumours from the same individual, a young girl, and the operation succeeded perfectly; but the wounds were a long time in cicatrising, and left scars, more or less marked; the cicatrices were a little deformed. This fact I have frequently remarked; and it is well to be aware of it as a guide in the prognosis of this kind of operations. After what I have said upon the certainty that these organic spots on the skin are the germs of sanguine tumours of more or less gravity, it is easy to conceive that it is important to get rid of them as soon as possible. Children should be operated on early in life, in order to prevent consecutive tumours; and this can be accomplished in various ways, as by caustic, continued methodical compression, and by a cutting instrument. [M. Roux here mentioned the case of his own daughter who, when quite young, had a mark as large as a finger nail on her right temple, and which was cured by methodical compression, continued for a long time; the spot gradually disappeared; all that remained was a little circle, somewhat darker than the surrounding skin, but which disappeared entirely later. She was entirely cured, and reached the age of twenty-seven years without any return.]

The vermilion red spots you see are generally arterial in their nature, and give rise to arterial sanguine tumours, whilst the black spots are formed by the veins, and produce venous tumours.

When we consider the important connection between these tumours and consecutive sanguine tumours, it is evident that we cannot be in too great haste to remove them, especially as at their commencement the treatment is very simple and easy, it consisting in removing a small piece of the skin, an operation which presents no difficulty.

In general *nævi materni* present themselves in the form of flat spots rather than of prominent tumours, of various colours. It is from this kind of spots that sanguine tumours owe their origin. But this principle should not be taken in too absolute a sense. There are spots, a little above the level of the skin, which in time finish by becoming sanguine tumours also. I shall now give you some examples of this kind, in order that what I have said may be the better engraved on your memory. Deschamps, who was Surgeon in Chief of La Charité, before Boyer, had on his leg, since his birth, a nævus maternus, somewhat prominent, and having none of the characters of those spots, which give rise to sanguineous tumours. However, when he was advanced in life, and perhaps from the effect of friction, it became soft and fungous, acquired a considerable volume, and from time to time split, giving rise to considerable hemorrhages. Had it been a young person, an insignificant operation might have been performed which would have ridden him of this infirmity, but the great age of Deschamps prevented the surgeons, his friends, from entertaining this idea. A lady of the ancient and illustrious family of Montmorency, had upon the head a small congenital tumour, to which, when young, she paid no attention, but which enlarged as she advanced in life, and presented a well marked arterial pulse, and gave rise to severe hemorrhages. Had it not been for those about her, I should, in spite of her age, have insisted upon removing it by an operation, and I believe much advantage would have ensued from such a step, as she died a short time after, and I believe that this small tumour upon her head was not without its influence upon her death.

There are then besides true *nævi materni* other organic alterations of the skin, which can later transform themselves into genuine sanguine tumours, and require extirpation, or some other treatment. Some pathologists have also admitted that congenital spots may sometimes be developed in the thickness of the skin itself, in the cellular tissue. Wardrop, who has occupied himself particularly with this subject, has admitted *sub-cutaneous nævi materni*, which are developed in the sub-cutaneous cellular tissue, and in other parts. I have seen them in the mucous membranes only; as these membranes are of a rose colour more or less lively, the spots upon them are seen with difficulty. We had an example in the young person we shall operate on this morning. Besides a very evident varicose tumour of the lower lip, she has also a small tumour of the same nature upon the point of the tongue, and upon the deepest part of this lip, which is but scarcely visible. Some years since a lady from Bordeaux came to Paris, recommended to me, and who had a sanguineous tumour at the inferior part of the rectum; it was as large as a small apple, but was happily pediculated, although the pedicle was as large as the index finger. This tumour, closing up the anal orifice, interfered considerably with defecation, and produced general disturbance in the functions of the intestines. It was essentially arterial, pulsated strongly, having in fact all the characters of those tumours which are called now-a-days erectile; on account of its pediculated form I employed a ligature. I used two ligatures, embracing in each one-half of the tumour. The patient was completely cured. The germ of this tumour was probably one of those congenital spots of which I have spoken, and which on account of its position had not been perceived.

These tumours then may be situated on the surface of the skin, as well as in the deepest and most hidden portions of the organism; in the substance of the bones they have been frequently seen, and they have been called aneurisms of the bones. Scarpa, in his beautiful work on aneurism reports a remarkable case situated in the thickness of the superior portion of the tibia. I have also observed a sanguine tumour of this nature in the radius of an individual. I felt the pulsations through a thin lamina of the osseous substance, in the midst of which it was developed. I treated it first by a ligature of the brachial artery. I will here tell you by anticipation that this means of treatment, viz.: a ligature on the principal artery of the limb, never succeeds. As much as this method is useful in cases of genuine aneurisms, so does it fail in sanguine fungous tumours. In this case the ligature of the brachial artery failed, and we were obliged to resort to amputation of the limb.

Let us now study, in a few words, the essential differences which exist between these tumours themselves. Some in their evolution follow a course analogous to encephaloid tumours, and are difficult to distinguish from them. There are some which are essentially formed by the arteries, (as I have already told you,) and others by the veins; to the formation of others both orders of sanguine vessels concur. When J. L. Petit was occupying himself with this species of tumours, he admitted, in general, in the beautiful description he gave of them in his works, the veins only as constituent elements. This is erroneous, for there are many tumours which have no other constituent elements but the arteries; we must, therefore, believe that this great surgeon had only occasion to study those sanguine tumours called *venous*, or *varicose;* but these are only one species, and not the whole family of these important affections.

I find in the same way the term *aneurism by anastomosis* imperfect, a denomination which the English have given to certain aneurismal tumours, and which I believe to be better defined under the name of aneurismal varices. I discussed this question thoroughly in the account of my trip to London, in 1814, and still more so in a memoir which I published on the subject in the Dictionnaire de Medecine et Chirurgie Pratiques. I shall not stop here. In spite of these works and others, which have been published, there is much yet to do; it is a very rich mine for study. I have not yet seized perfectly well, for example, the differences between sanguine tumours, and false aneurisms.

The tumour of our little patient is essentially venous; it is not difficult to recognize it, and to distinguish it from arterial sanguine tumours. In these, (as I have already observed,) there are pulsations, synchronous with those of the arteries, propagating themselves to the principal vessels of the arterial system of the region, branches which concur to nourish the tumours by their blood. It is on account of these characters that this variety of tumours are called aneurismal, having in reality characters in common with this last affection.

[Here M. Roux presented several designs representing sanguineous tumours of the face, upon which he had operated in the course of his practice. He showed, 1st. The portrait of a little girl with a sanguineous tumour seated upon the right side of the nose, and invading a portion of the corresponding cheek, as far as its inferior portion. 2d. A design representing a young girl with a considerable venous sanguineous tumour of the superior lip, which extended to the middle portion of the right cheek; she was treated by means of needles thrust into the tumour, around which ligatures were passed.]

I have treated, said M. Roux continuing, another similar case in a young girl, and cured it by ligatures of the coronary, supraorbitar and other arteries, which reduced greatly the extent of the tumour, and I removed by excision what was left, which was of but little volume. Here is a drawing in which you see a fungous tumour developed in the right orbit, projecting under the superior eyelid, cured, at least by five-sixths, by means of a ligature to the primitive carotid. This region of the face is subject to this kind of tumours, the reason for which is not known. I have seen, for my part, a very large number. In this patient there was much that was really curious; he was somewhat savage, and uncivilized, (for, although of a certain station, he had always lived alone in the woods, isolated from society,) and would never submit to the hygienic prescriptions I gave him, after the ligature of the vessel; he went about, walking and eating as usual; no accident, however, happened to hinder the natural effects of the operation. The tumour diminished, as I said, by five-sixths, but the patient would go, and at his departure it still pulsated a little.

*Treatment.*—We are now-a-days much richer in therapeutic and operative means against these affections, than formerly; all these methods can be arranged in four groups, applicable to the several indications. The first which suggested itself to the mind, was to remove the tumour by means of a cutting instrument; here we acknowledge the necessity of carrying the incision beyond the limits of the disease, if a return is not wished for. J. L. Petit has strongly expressed this indication; he greatly insists on cutting the tissues, where the condition of the vessels is normal. This method, though still employed, is much less so than formerly, for other methods replace it in a number of cases. I shall return to it presently.

In the second may be placed all those means which will cause the disappearance of the tumour by pressure. This is sometimes very useful, and will cause them to disappear entirely; but they should not be too large, and they should rest upon a subjacent osseous surface, because compression is only efficacious when acting on a solid and resisting surface, and it is necessary too that it be continued for a long time. By the aid of this means the enlarged vessels gradually regain their former dimensions; a certain degree of inflammation takes place in the tissues, and transforms them into new tissues as it were, thus destroying the disease. In 1812, during a voyage that I made into Spain, a child was born in a family, with which I was intimately connected, with a blood mark on her breast. On my return to Paris the mother requested me to see the child immediately, as she was very uneasy about the mark, and its future character. Already it had increased in volume and formed a little tumour, situated just above the nipple. Before undertaking anything, as it cost me a great deal to use a cutting instrument, I wished to have the advantage of the opinion of Boyer. We agreed upon the utility of compression. I had a little plate made expressly, which, by means of a spring, could apply itself upon the whole tumour, and compress it within certain limits. In spite of this methodical and regular compression, and being well watched by me, the tumour increased, and pulsations were developed in it so strong, that they could be felt in the arterial branches of the axilla. I became very uneasy, for I could not foresee the result. I then conferred with Boyer and other surgeons, and they were of opinion that the compression had better be removed, and that we should wait awhile. The tumour, left to itself, remained sometime stationary, then commenced to diminish, and in spite of the arrival of puberty, the period so often favorable to the developement of these kinds of tumours, continued constantly to diminish; and finished by completely disappearing, and was never reproduced. The individual is now married, and has children; I see her frequently, and I know positively that the cure of the tumour was radical. I designedly insist upon this circumstance, for the journals of the time, and some surgeons who published the fact, seemed to think that the disease reappeared later; which is entirely false.

In the third series we arrange all the means relating to ligature of the tumour, whether general, partial, or by needles, or by simple threads, etc. We now use the ligature entire, or have recourse to needles, by the aid of which you embrace a portion only of the tumour at the time. It has been thought, that you could advantageously tie the principal arteries which nourish the sanguine tumour, in the same way as in the treatment for aneurism. They have employed the method Hunter proposed for aneurisms; but it very often failed, especially in those cases of sanguine tumour, developed in the members; this method, on the contrary, seemed to succeed in those tumours developed in the orbit.

Sometimes we are obliged to eradicate sanguineous tumours, which have roots, by means of which they irradiate themselves here and there into the tissues. This is a good method whenever you can practise it without too much difficulty, and destruction of the surrounding parts. Still, however, we have been too long under the empire of the ancients in this regard, principally J. L. Petit. There was a little exaggeration on the part of this great surgeon, for experience proves they do not grow again near as often as those of other kinds, even when a trace of their presence is left in the tissues; ordinary suppurative inflammation takes place in these roots, and the tissues return to their normal condition. Ligature of the primitive carotid, for example, is indicated for those sanguine tumours situated in the orbit, because it is impossible to reach directly the vessels which surround and nourish the tumour. I have often given an explanation of this result, which has been adopted by authors and journals; this is it: Tumours which are developed in the orbit ought to have as their immediate source, or principal cause, the action of the ophthalmic artery, or some one of its branches; in tying the primitive carotid, you arrest, first, the flow of blood in the ophthalmic artery: now this artery constitutes a little arterial system apart; it has no communication with the other arterial systems, and loses itself, so to speak, spending itself in the eye and its appendages, so that the carotid artery being tied, the blood cannot easily enter the eye; consequently there is a great deal of chance of arresting the blood which flows in the tumours; which does not happen in the sanguineous tumours of the members, and which causes the ligature of the principal vessels communicating with the tumour to fail so often; for in this case the ligature of the principal vessels does not hinder the blood from circulating in the collateral branches of the affected member, and thus continue to nourish the tumour. Eight years ago a case worthy of note presented itself to me. A young girl had a sanguineous tumour of the epigastric region, placed in the midst of the muscles of that region, so that it seemed to be nourished immediately by the internal mammary and epigastric arteries. We then thought of treating it by a ligature of these two arteries. If it were now, I should greatly hesitate before adopting a similar treatment.

In the last group are placed all those various methods lately introduced into the science, and by means of which you are enabled to destroy the tumour without a bloody operation. This method is to produce a peculiar inflammation in the tumour capable of transforming it into a different tissue comparatively normal. I was the first to propose this method. It consists of cauterization, especially recommended by Wardrop, and the introduction of needles into the tumour, proposed by M. Lallemand, and followed with much success. The inflammation resulting from the employment of this method, causes softening in the diseased tissue; then a peculiar friability; then, the inflammation subsiding, a condensation of the tissues results, belonging to the natural condition.

All these methods have great advantages, and at the same time some inconveniences, so that you must understand the precise indications to employ them with advantage.

To no one of these methods, at present, can preference be given over the others, because they have not been employed long enough to enable us to form any comparative estimation of their value. M. Lallemand lauds greatly his needles and their ligatures; he assures us that he has obtained numerous and splendid results, and I believe him, because I have myself employed them with advantage; but still sufficient time has not yet elapsed since the employment of the method, for it to be definitely judged.

The same reflections may be made in regard to cauterization; I have tried it; it has sometimes succeeded, and sometimes failed.

It has also been proposed'to tear up the cavities of the tumours, followed by an injection of an irritant liquid into these artificial cavities. By this method I am always fearful of producing too much inflammation, and I do not ordinarily employ it. You can besides multiply infinitely the operative methods, and means of treatment against this description of affection, but it is better to keep to those which are already known and have been submitted to the test of experience.

I shall now give you a summary of the case of the young girl that I am going to operate on. She is eighteen years of age, habitually of good health, and regular. From birth she had a small bluish tumour upon the lower lip. When quite young, it was perceived that her tongue was thicker on one side than the other, and presented at this point a bluish colour. The disease increased, though slowly, and now there is a tumour occupying two-thirds of the left side of the lower lip, and particularly the mucous membrane, which is pushed in front, and downwards, so that the cutaneous portion of the lip rests upon the chin. When the patient is quiet the tumour is about the size of the thumb; its surface is bluish-red, somewhat uneven and wrinkled; it is soft, and can be entirely reduced by compression. There is no pulsation in it. If, on the contrary, the patient makes any effort, which momentarily suspends her respiration, or throws her head forward, the size of the tumour doubles, equalling in size the egg of a pigeon; its colour becomes of a deep violet; it is shining, tense, without inequalities or wrinkles; resists pressure, but no pulsations are felt. The patient returning into a state of repose, the tumour regains its original dimensions. This affection reaches to the posterior surface of the lip, as far as the gums, and extends on the left side to the internal portion of the cheek. The left half of the tongue is similarly affected, but during exercise its developement is not so great; speech and deglutition are not sensibly impeded.

[On the 3d of May, M. Roux operated, by thrusting through the tumour of the lip on the mucous side, several pins, surrounding them with threads; each ligature comprising the greatest thickness of the lip. He passed three times a red hot stylet horizontally into the tumour. After the operation the tumour swelled, and became tense and shining.

On the 10th, the pins being moveable, were withdrawn. The tumour was more resisting, and had acquired the consistence of flesh; its volume, however, was but slightly diminished. Towards the 18th, it became somewhat softer.

On the 9th of May, M. Roux determined to pass three setons through the base of the tumour of the lip with the same thread, which he introduced three times zig-zag from within outwards, and from without inwards. For several days afterwards the size of the tumour increased, and it became more resistant than before the operation. On the 13th of June a slight venous hemorrhage occurring, a ligature, including the free border of the lip to the internal margin of the tumour was applied, which was removed on the 15th, which had cut through a portion of the tissues, strangulated by the thread.

On the 9th of July M. Roux, seeing that the tumour was very much reduced in size, determined on removing a large part of it by means of a cutting instrument. He accordingly, with a pair of scissors, removed at a single sweep the whole portion contained within the mouth, leaving a mucous border to the extent of a centimetre, so as to have an ordinary lip. A great deal of venous blood was lost, the wound was stuffed with charpie and compressed against the dental arch. On the 10th of July the dressing was removed, and the wound found finely suppurating. The suppuration gradually diminished; cicatrization advanced; the wound was cauterized with the nitric acid of mercury. The lip was reduced to the same size as its fellow, the violet colour alone remaining. She was discharged on the 26th of August.]

*Paris, August*, 1843.

